# Field-Dependent Resonant Behavior of Thin Nickel Film-Coated Microcantilever

**DOI:** 10.3390/mi8040109

**Published:** 2017-04-01

**Authors:** Yunhee Park, Eun Joong Lee, Taejoon Kouh

**Affiliations:** Department of Physics, Kookmin University, Seoul 136-702, Korea; dbs3766@kookmin.ac.kr (Y.P.); lej80645@kookmin.ac.kr (E.J.L.)

**Keywords:** microcantilever, mechanical resonance, thin magnetic film, magnetostriction, surface stress

## Abstract

Herein we describe the vibration of a thin nickel film-coated microcantilever at resonance under an external magnetic field. The resonance frequency and the mechanical loss—experimentally observed while varying the magnetic field—closely follow the field-dependence of the magnetostriction coefficient, indicating the strong coupling between the mechanical motion and the magnetostriction through the surface stress. Comparing to the surface stress model based on uniformly distributed axial load, the magnetostriction coefficient of a nickel film has been estimated, and its value is comparable to the reported one. Our study suggests that the nature of the surface stress originating from the magnetostrictive film can govern and modulate the resonant behavior of miniaturized mechanical systems.

## 1. Introduction

Magnetism has played a key role in human history, from ancient navigation to modern medical imaging. Among the various related effects, magnetostriction has helped to open up the early stage of the technological advances through the magnetomechanical interaction [1]. However, due to the small coupling stress, manifested as the magnetostriction coefficient (typically on the order of a few millionths), its application to modern engineering problems is rather limited. This has led to continuing works on magnetostrictive materials, developing magnetic compounds with extremely large magnetoelastic response [2,3]. Interestingly, miniaturized mechanical systems can provide a means to re-exploit this magnetostriction effect at small scales, since these show considerable responses even under small external perturbation [4]. Attempts to couple magnetic systems to the tiny motion of miniaturized vibrating mechanical structures have been reported; for example, showing the investigation of magnetic material characteristics [5,6] and the sensitive detection of an external magnetic field [7,8]. Such works prove that a sufficient reaction of miniaturized mechanical systems can be anticipated from the interaction with magnetic systems, which in turn can offer viable metrological tools to investigate a variety of magnetic phenomena.

With these in mind, this manuscript focuses on the magnetostriction effect in a miniaturized mechanical structure, particularly at its vibration mode, while many of the previous related studies have relied on the static deflection/bending of a cantilever beam structure and the possible integration with magnetic materials exhibiting giant magnetostriction effect [6,7,9,10,11]. For the experiment, the dynamic response of a microcantilever—a widely-used scaled-down mechanical resonator—with a very thin layer of magnetic nickel film on top is observed under external magnetic field. To understand the experimentally-observed change in the resonant response of the microcantilever originating from the magnetostriction effect, the theoretical description of surface stress effect on the cantilever structure is also considered. The presented work demonstrates the feasibility of magnetomechanical coupling in the mechanical structure through a very thin magnetostrictive layer. It also suggests that the induced magnetostriction effect via external field can help to examine the fundamentals of surface mechanics in a small structure.

## 2. Experiment

**Figure 1:**
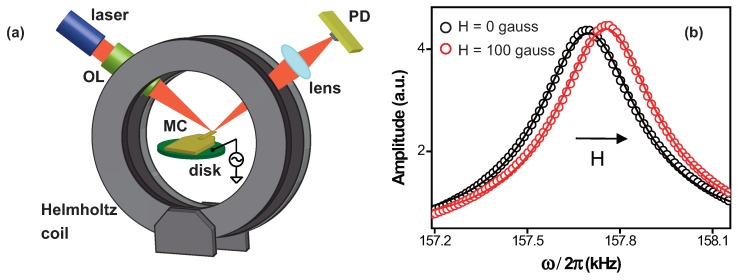
(**a**) Schematic of the experimental set-up. The Ni film-coated microcantilever (MC) was placed on a piezoelectric disk inside of a Helmholtz coil. The laser was focused on the tip of the cantilever with an objective lens (OL) and reflected back to a segmented photodetector (PD) through a focusing lens. The magnetic field was applied in the direction parallel to the microcantilever; (**b**) the frequency spectrum of the 40 nm-thick Ni film-coated microcantilever near resonance before and after applying magnetic field of 100 gauss. The solid lines represent the Lorentzian fit to each spectrum. One can notice the shift of the resonance frequency under the external magnetic field.

For the experiment, thin nickel (Ni) films with thickness of 20, 40, and 60 nm were deposited on a commercial silicon (Si) microcantilever having a dimension of *l* × w×t = 225 μm ×38 μm × 7 μm, by the resistive thermal evaporation technique from a Al${}_{2}$O${}_{3}$ crucible at a base pressure of around $10^{-7}$ Torr using a high-purity nickel (99.995 %) source at a rate of 1 Å/s at room temperature. The thickness of the Ni film was monitored with a quartz crystal microbalance during the thermal deposition process. After the deposition, the Ni film-coated microcantilever was placed on a piezoelectric disk inside of a Helmholtz coil, which can generate a direct current (DC) magnetic field up to ±100 gauss. The frequency spectrum of the microcantilever around the resonance was measured at room temperature in air by the optical beam deflection technique [12] under the external magnetic field. The light was focused on the microcantilever through an objective lens, and the reflected light from the surface was refocused onto a segmented photodetector. The modulation of the optical signal from the photodetector due to the change in the position of the optical spot on the photodetector was monitored while actuating the cantilever by applying alternating current (AC) voltage to the piezoelectric disk and varying the magnetic field along the direction parallel to the cantilever length, as shown in Figure 1a. We also deposited a Ni film with identical thickness on a glass substrate at the same time as the deposition was done for the microcantilever and measured the room-temperature magnetic hysteresis curve with a vibrating sample magnetometer (VSM, Lakeshore Model 7404, Westerville, OH, USA).

## 3. Results and Discussion

Figure 1b displays the effect of the magnetic field on the frequency spectrum of the 40 nm-thick Ni film-coated microcantilever near resonance without and with magnetic field of 100 gauss. Under zero magnetic field, the microcantilever showed the resonance frequency of $\omega_{0}/2\pi ∼$ 157.70 kHz with a quality factor of Q∼ 396 based on the full width at half maximum (FWHM) of the frequency spectrum. Under a DC magnetic field of 100 gauss, $\omega_{0}/2\pi$ changes to 157.75 kHz and *Q* of 400. The measurement uncertainties in determining both frequency and quality-factor were 0.2 Hz and 0.6, respectively. This shows that the external magnetic field affects the resonant behavior of the Ni film-coated microcantilever.

**Figure 2:**
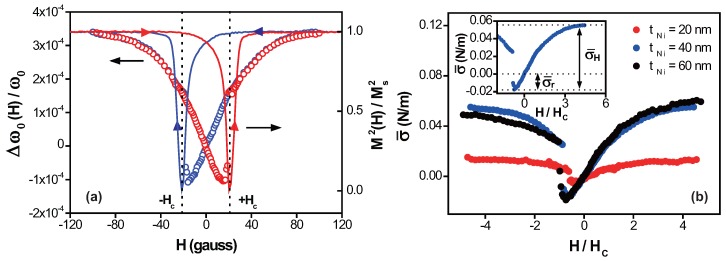
(**a**) Frequency-shift curve $\Delta \omega_{0}\left(H\right)/\omega_{0}$ as a function of the external magnetic field *H* between −100 and +100 gauss of 40 nm-thick Ni film-coated microcantilever, shown as open circles. The red circle represents the upward magnetic field sweep direction and the blue circle represents the downward direction. The solid lines are the normalized square of magnetization ${\left(M\left(H\right)/M_{s}\right)}^{2}$ curve of 40 nm-thick Ni film. Again, the red line indicates the upward sweep direction, and the blue is for the downward direction. The error bar is same as the size of the symbol. The coercivity field $H_{c}$ is indicated with the dotted line; (**b**) surface stress $\bar{\sigma }$ as a function of the normalized magnetic field $H/H_{c}$ for 20, 40, and 60 nm-thick Ni film-coated microcantilevers, as the field sweeps in the downward direction. The shape of the $\bar{\sigma }$-curve for the upward direction is identical, except for the jump in the surface stress occurring at $H/H_{c}\approx$+1, as expected from the frequency-shift curve in (a). The inset illustrates the field-independent residual stress ${\bar{\sigma }}_{r}$ and the magnetic field-dependent surface stress ${\bar{\sigma }}_{H}$ for $t_{Ni}$ of 40 nm.

Since the typical soft ferromagnetic materials exhibit weak magnetoelastic response (known as the ΔE effect [13]), we believe that the magnetostriction-induced surface stress in the top Ni layer under external field is responsible for the observed resonance frequency shift [14]. Figure 2a shows the amount of frequency shift between zero-field and non-zero-field resonance frequency, $\Delta \omega_{0}\left(H\right)/\omega_{0}=\left(\omega_{0}\left(H\right)-\omega_{0}\left(H=0\right)\right)/\omega_{0}\left(H=0\right)$, as the magnetic field *H* is swept between −100 and +100 gauss. To compare the observed frequency shift with the magnetic property of the Ni film, the square of the magnetization, ${\left(M\left(H\right)/M_{s}\right)}^{2}$, normalized with the saturation magnetization $M_{s}$ is included in Figure 2a. There are two distinct features apparent in the frequency-shift curve $\Delta \omega_{0}\left(H\right)/\omega_{0}$. One is the hysteresis in the curve depending on the magnetic field-sweep direction, reaching the saturation value above around ±80 gauss. The other is the sudden jumps in frequency at the magnetic field, corresponding to the coercivity field $H_{c}$, which is about 22 gauss for a 40 nm-thick film: a jump at −22 gauss in the downward sweep direction and a jump at +22 gauss in the upward direction. The close resemblance between these curves can be expected, as the magnetostriction coefficient λ(H) varies as $3\lambda_{s}M{\left(H\right)}^{2}/2M_{s}^{2}$, where $\lambda_{s}$ is the saturation magnetostriction [1]. Since the film studied is polycrystalline without preferred grain orientation [15], the magnetostriction coefficient considered in this work is the averaged value over the crystallographic directions.

To further clarify this magnetostriction effect in the cantilever frequency shift, we turn to the surface stress model proposed by McFarland et al. [16] based on uniformly distributed axial load. In this model, for a small frequency shift, the relation between the frequency shift and the surface stress $\bar{\sigma }$—defined as axial force per a unit length—is given as

(1) $$\bar{\sigma }\left(H\right)=2\frac{\Delta \omega_{0}\left(H\right)}{\omega_{0}}\frac{Ewt^{3}\pi^{2}}{24l^{3}}$$

where *E* is the Young’s modulus. Here, the compressive surface stress—taken as $\bar{\sigma }>0$—will tend to stretch the cantilever with increasing frequency, and the tensile stress of $\bar{\sigma }<0$ will generate the opposite effect. The Ni film on top with a negative value of λ(H) undergoes the compressive deformation under the external field along the cantilever length, and is expected to show $\Delta \omega_{0}\left(H\right)/\omega_{0}>0$. In addition, by considering the cross-section of the rectangular cantilever and the strain–stress relation where the strain would be the magnetostriction coefficient in a magnetostrictive material, we expect $\bar{\sigma }\left(H\right)=-Ewt\lambda \left(H\right)/l$, which indicated that $\Delta \omega_{0}\left(H\right)/\omega_{0}\propto M{\left(H\right)}^{2}$, as seen in Figure 2a. Based on the experimentally observed $\Delta \omega_{0}\left(H\right)/\omega_{0}$ with Equation (1), the value of $\bar{\sigma }$ is calculated and shown in Figure 2b. For the composite cantilever used in this experiment, the effective value of Young’s modulus $E_{eff}=\left(E_{Ni}t_{Ni}+E_{Si}t_{Si}\right)/\left(t_{Ni}+t_{Si}\right)$, where $E_{Ni}$, $E_{Si}$, $t_{Ni}$, and $t_{Si}$ are the Young’s modulus [17] and thickness of Ni film and Si cantilever used for the calculation.

The overall calculated value of $\bar{\sigma }$ and the corresponding $\Delta \omega_{0}\left(H\right)/\omega_{0}$ are positive, but we notice the appearance of negative $\bar{\sigma }$—associated with the decrease in $\omega_{0}\left(H\right)$—near $\pm H_{c}$. If the change in the surface stress is solely coming form the magnetostriction effect, we expect the minimum value of $\bar{\sigma }$ would be zero, since $\bar{\sigma }\propto {\left(M\left(H\right)/M_{s}\right)}^{2}$. We believe that this is due to the presence of the residual stress (resulting from the thermal deposition of Ni film on top of Si cantilever as well as the intrinsic stress in the bare cantilever), and the negative sign indicates that this is tensile in nature. Therefore, the total surface stress $\bar{\sigma }\left(H\right)$ determined from the experiment has two contributions of field-independent residual stress ${\bar{\sigma }}_{r}$ and the magnetic field-dependent surface stress ${\bar{\sigma }}_{H}\left(H\right)$, originating from the magnetostriction effect (as indicated in the inset of Figure 2b). The values of ${\bar{\sigma }}_{r}$ and ${\bar{\sigma }}_{H}^{s}$—the saturation value measured at $H/H_{c}\approx 5$—are shown in Table 1. The corresponding Ni film stress—in units of N/m${}^{2}$—is on the order of 10${}^{6}$ N/m${}^{2}$, which agrees with the film stress observed in thin Ni film, induced by magnetostriction [18]. The saturation magnetostriction coefficient $\lambda_{s}$ of Ni film can now be determined from ${\bar{\sigma }}_{H}^{s}=-3E_{eff}wt\lambda_{s}^{eff}/2l$, where $\lambda_{s}^{eff}$ is the effective saturation magnetostriction coefficient of the cantilever, defined as $\lambda_{s}^{eff}=\lambda_{s}E_{Ni}t_{Ni}/\left(E_{Ni}t_{Ni}+E_{Si}t_{Si}\right)$. The value of $\lambda_{s}$ obtained for each film thickness is also shown in Table 1, with an average value of $-2.7\pm 0.9\times 10^{-5}$. This is close to the reported value of $\lambda_{s}$ for Ni, which is around $-3.4\times 10^{-5}$ [19].

**Figure 3:**
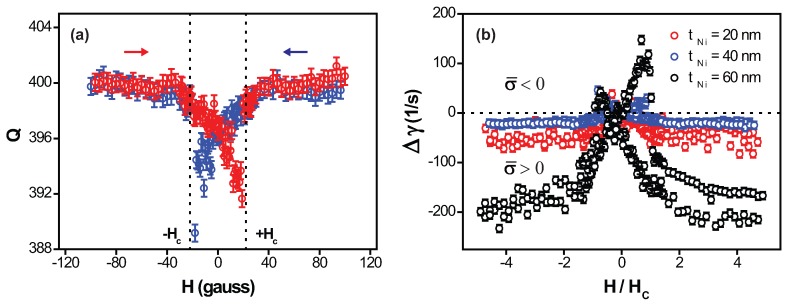
(**a**) The quality factor *Q* of 40 nm-thick Ni film-coated microcantilever as a function of the external magnetic field *H* between −100 and +100 gauss. The field-dependent *Q*-curve shows hysteresis similar to the frequency-shift curve, depending on the sweep direction. Again, the red circle represents the upward magnetic field sweep direction and the blue circle represents the downward direction; (**b**) change in dissipation constant, Δγ(H), as a function of normalized magnetic field, $H/H_{c}$, while the magnetic field is swept in both directions. For $-H_{c}<H<+H_{c}$, Δγ(H) is positive, which can be related to $\bar{\sigma }<0$, while Δγ(H) is negative, associated with $\bar{\sigma }>0$ for $H<-H_{c}$ and $H>+H_{c}$.

From the resonance spectrum, we have also determined *Q*-factors as a linewidth of the spectrum while varying the magnetic field, and these are shown in Figure 3a for $t_{Ni}$ of 40 nm. The *Q*-factor curve exhibits a similar field-dependency as in the frequency-shift curve: hysteresis associated with the field sweeping direction along with jumps near the coercivity field and reaching a saturation value when $H>H_{c}$. This indicates the occurrence of the energy dissipation within the cantilever induced by the magnetostriction effect. Since the magnetostrictive Ni film gives rise to changes in both frequency and linewidth, in order to see the level of the energy loss under the external field, we consider the dissipation constant $\gamma \left(H\right)=\omega_{0}\left(H\right)/Q\left(H\right)$. Figure 3b displays the change in dissipation constant, $\Delta \gamma \left(H\right)=\gamma \left(H\right)-\gamma_{0}$, as a function of normalized magnetic field, where $\gamma_{0}$ is the value of γ at zero magnetic field. Within $-H_{c}<H<+H_{c}$, the increase in dissipation is observed, while the reduction in the dissipation can be seen for $H<-H_{c}$ and $H>+H_{c}$. As in Figure 2b, each region of *H* can be characterized with the surface stress as the compressive surface stress ($\bar{\sigma }>0$) for $H<-H_{c}$ and $H>+H_{c}$, and tensile surface stress ($\bar{\sigma }<0$) for $-H_{c}<H<+H_{c}$. This suggests the effect of the stress on the mechanical loss within the cantilever, as observed from the high *Q* value of an oscillating mechanical structure under high stress [20]. However, we notice quite a large variation in Δγ(H) between different Ni film thicknesses. For example, the value of $\Delta \gamma (H\approx 5H_{c})$ for $t_{Ni}$ = 60 nm is about 10 times larger than the value for $t_{Ni}$ = 40 nm, despite the fact that these two show comparable values of $\bar{\sigma }$ and the film thickness only differs by a factor of 1.5. Additionally, at zero magnetic field, these cantilevers show rather close values of initial dissipation constant—$\gamma_{0}$ of 2549 s${}^{-1}$ for $t_{Ni}$ = 20 nm, 2500 s${}^{-1}$ for $t_{Ni}$ = 40 nm, and 2584 s${}^{-1}$ for $t_{Ni}$ = 60 nm. These suggest that the mechanical energy loss might be influenced by other factors, which are not clearly identified in this work. Some of the possibilities are that the surface friction which these thermally-evaporated magnetostrictive films experience might be sample-dependent, with deviations in the surface morphology and inhomogeneity in film, since the surface effect in the miniaturized mechanical resonators has been well-known for its crucial role in the mechanical dissipation [21,22,23]. We are not currently sure of the exact origin of this variation in Δγ(H), but further work on this might be found interesting, possibly leading to detailed understanding of surface mechanics in micro-/nanoscale mechanical resonators.

## 4. Conclusions

In conclusion, we have investigated the dynamics of a thin magnetostrictive Ni film-coated microcantilever under external field at the mechanical resonant mode. The experimentally-observed behaviors of both frequency shift and the mechanical dissipation closely follow the field dependency of the square of magnetization curve—hysteresis and abrupt changes near the coercivity field—describing the magnetostriction effect, and agree with the uniform axial load model. This suggests that the magnetostriction-induced surface stress is strongly coupled to the mechanical vibration, allowing the careful examination of the surface mechanics in miniaturized mechanical systems.

## Figures and Tables

**Table 1:** Coercivity field $H_{c}$, residual surface stress ${\bar{\sigma }}_{r}$, field-dependent saturation surface stress ${\bar{\sigma }}_{H}^{s}$ (which is measured at $H/H_{c}\approx 5$), and saturation magnetostriction coefficient $\lambda_{s}$ for Ni film thickness of $t_{Ni}$.

$t_{\mathrm{Ni}}$ (nm)	$H_{c}$ (gauss)	${\bar{\sigma }}_{r}$ (N/m)	${\bar{\sigma }}_{H}^{s}$ (N/m)	$\lambda_{s}$
20	21.2	$-5\times 10^{-3}$	$1.8\times 10^{-2}$	$-1.8\times 10^{-5}$
40	21.8	$-1.8\times 10^{-2}$	$7.3\times 10^{-2}$	$-3.6\times 10^{-5}$
60	20.4	$-1.9\times 10^{-2}$	$7.8\times 10^{-2}$	$-2.6\times 10^{-5}$

## Abbreviations

The following abbreviations are used in this manuscript:

AC	Alternating current
DC	Direct current
FWHM	Full width at half maximum
Ni	Nickel
*Q*	*Q*-factor: quality factor
Si	Silicon
VSM	Vibrating sample magnetometer
